# Obesity and Lifespan Health—Importance of the Fetal Environment

**DOI:** 10.3390/nu6041725

**Published:** 2014-04-24

**Authors:** Alice F. Tarantal, Lars Berglund

**Affiliations:** 1 Department of Pediatrics, School of Medicine, University of California, Davis, CA 95616, USA; E-Mail: aftarantal@ucdavis.edu; 2 Department of Cell Biology and Human Anatomy, School of Medicine, University of California, Davis 95616, CA, USA; 3 California National Primate Research Center, University of California, Davis, CA 95616, USA; 4 Department of Medicine, School of Medicine, University of California, Davis, CA 95616, USA; 5 Department of Veterans Affairs, Northern California Health Care System, Sacramento, CA 95817, USA

**Keywords:** adipose tissue, pregnancy, metabolic syndrome, birth weight, risk factors, fetal environment, lifespan health

## Abstract

A marked increase in the frequency of obesity at the population level has resulted in an increasing number of obese women entering pregnancy. The increasing realization of the importance of the fetal environment in relation to chronic disease across the lifespan has focused attention on the role of maternal obesity in fetal development. Previous studies have demonstrated that obesity during adolescence and adulthood can be traced back to fetal and early childhood exposures. This review focuses on factors that contribute to early developmental events, such as epigenetic modifications, the potential for an increase in inflammatory burden, early developmental programming changes such as the variable development of white *versus* brown adipose tissue, and alterations in organ ontogeny. We hypothesize that these mechanisms promote an unfavorable fetal environment and can have a long-standing impact, with early manifestations of chronic disease that can result in an increased demand for future health care. In order to identify appropriate preventive measures, attention needs to be placed both on reducing maternal obesity as well as understanding the molecular, cellular, and epigenetic mechanisms that may be responsible for the prenatal onset of chronic disease.

## 1. Introduction

The societal burden of chronic diseases continues to rise [1] representing a serious health care challenge. The increase in disease burden is not limited to any particular segment of the population but is seen across ages, race/ethnicity groups, and gender, as exemplified by the high prevalence of the metabolic syndrome and related long-term consequences [2]. In particular, attention has focused on the increase in childhood obesity and its underlying causes [3]. Childhood obesity has been suggested to contribute to an alarming shift in the childhood health spectrum from acute to chronic illnesses with long-term negative effects. Interestingly, prenatal conditions have been proposed to be associated with an increased risk of cardiovascular, renal, and metabolic diseases later in life [4,5,6,7,8], suggesting that the fetal environment sets the stage for future susceptibility to chronic diseases. An increase in obesity among pregnant women has been linked to this finding [9,10,11], indicating that the focus of preventive effects may span the generations. Management of chronic diseases over the lifespan is likely to consume an ever increasing fraction of economic resources devoted to health care [12], and efforts to address underlying causes of common chronic diseases are therefore of critical importance. Despite the growing body of published data, the specific mechanisms mediating early programming remain elusive and the potential “windows of susceptibility” have not been well defined. 

The importance of this growing subject of national concern was highlighted in the NIH Common Fund Strategic Planning for 2013 as “*Developmental Origins of Health and Disease: Disease Prevention across Generations*”. The developmental origins of disease hypothesis proposes that organ systems undergo programming prenatally, which sets the stage for physiologic and metabolic adaptations during later life [4,5,6,7]. Although this hypothesis has been primarily studied in the context of cardiovascular and metabolic disease, the continued rise in allergic and autoimmune diseases highlights a broader impact through the susceptibility of immune pathways [13,14]. The role of the immune system as a modulator of many disease manifestations across the lifespan is increasingly being recognized and inflammation is an acknowledged common theme for many chronic diseases [6,15,16,17]. It is notable that obesity may promote a chronic inflammatory state, which in turn may lead to many downstream consequences, such as insulin resistance, resulting in a vicious cycle. Further, a range of factors has been shown to alter epigenetic programming and gene expression *in utero* that can have profound implications for the developing phenotype and disease predisposition [18,19,20,21]. A critical question to consider is to what extent exposure to an obese environment during fetal life with an ensuing impact on the developing immune system can predict outcomes leading to chronic disease conditions with maturation and aging. In the present review, we will focus on some potential mechanisms that can underlie and catalyze long-term disease consequences across the lifespan. 

## 2. Pregnancy and Maternal Obesity

**Figure 1:**
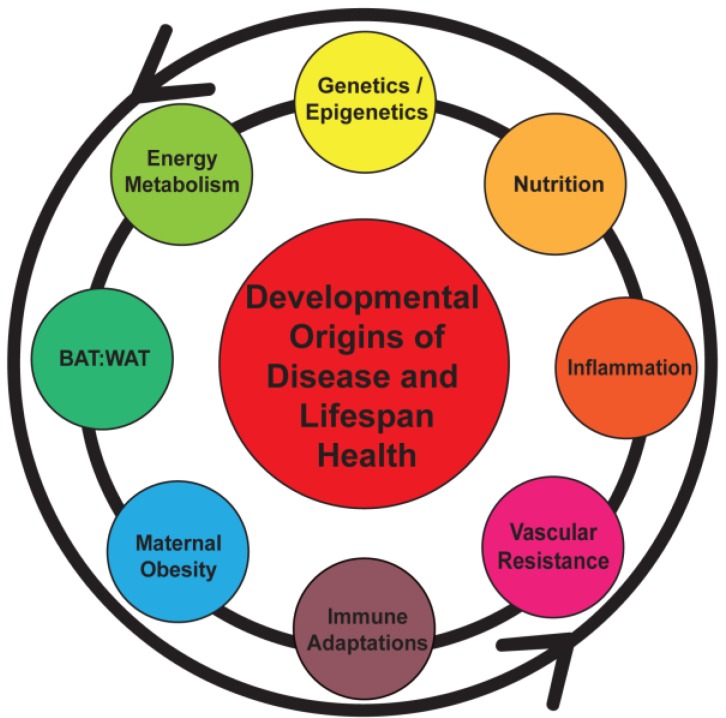
Early programming can be initiated by a number of maternal and fetal factors that sets the stage for a lifetime of health or disease. BAT, brown adipose tissue; WAT, white adipose tissue.

The number of overweight and obese individuals has increased worldwide, and the quantity of obese individuals surpasses the number of malnourished subjects. In the U.S., the population with an increased body mass index (BMI) has shown a steady rise over a 25-year period, and at present more than 65% of adults are classified as overweight and more than one-third are obese [9]. Should this continue, the notion of a normative weight range may become rare. This finding has resulted in classifying current findings as “the obesity epidemic”. An important consequence of this finding is an increasing number of overweight women of childbearing age. While many disease manifestations have been associated with obesity there is a lack of insight into specific pathways by which an environmental challenge such as maternal obesity might unleash changes in key molecular or cellular programming setting the stage for long-term health consequences. A better understanding of underlying mechanisms is critical to develop successful intervention strategies (Table 1, Figure 1).

**Table 1:** Potential mechanisms by which maternal obesity can impact fetal development and future health.

Mechanisms	References
Endocrine changes	[20,22,23,24,25,26,27,28,29,30,31,32,33,34,35,36]
Epigenetic modifications	[17,18,19,20,21]
Differential development of brown and white adipose tissue	[37,38,39,40,41,42]
Increased inflammatory burden	[22,23,29,31]
Immune system adaptations	[34,43]
Changes in vascular resistance and development	[22,29,32,44,45]
Ectopic fat accumulation	[28,46,47,48,49]
Nutritional modifications (e.g., fructose intake)	[30,50,51,52,53]
Energy metabolism	[54,55,56]

Studies indicate that an increased maternal BMI prior to pregnancy in addition to a large weight gain during pregnancy predicts obesity in offspring [9,22,44,57]. The issue of fetal weight as a predictor of lifespan health is more complex as epidemiologic studies indicate that both low and high birth weights are associated with later life disease; the former with accelerated growth postnatally as a “catch-up” phenomenon [9]. More extreme cases have been studied in the context of historical episodes of severe undernutrition during pregnancy, such as the Dutch famine during World War II and findings in Nigeria. Studies from these settings have demonstrated an increased presence of metabolic syndrome in adulthood [58,59]. The higher frequency of chronic disease linked with either maternal obesity or undernutrition suggests the presence of a U-shaped phenomenon with an “ideal” fetal weight that might optimize long-term health. However, the relationship between fetal growth and childhood/adult obesity appears to be continuous [9,46]. These observations have heightened the need to enhance our understanding of mechanisms that may develop and sustain the impact of an unfavorable fetal environment. 

Recent studies have focused on endocrine and metabolic factors associated with pregnancy, such as prenatal glucocorticoid excess and nutrition, inflammation, and maternal disease in an attempt to explain the high incidence of childhood obesity [22,23,24,25,26,27,28,29,30,31,32,33,34,57]. However, a firm understanding of the critical pathway(s) that result in lasting effects remains elusive, and this public health problem has not been adequately addressed. Lessons learned during such studies may have wider implications and represent a powerful driver of a future explosive health care demand. The striking trend towards obesity in childhood is a serious indication of major public health consequences and a lifelong burden of chronic disease on individuals and society.

## 3. Obesity, Chronic Inflammation, and Pregnancy

Adipose tissue is the largest endocrine organ in the body secreting hormones, cytokines (adipokines), and proteins that can substantially impact a wide variety of functions [35]. However, factors determining adipose tissue distribution and properties are not yet fully understood. In simple terms, obesity develops when the intake of calories exceeds energy expenditure. Depending on the time of onset, obesity is associated with increased adipocyte size and/or number. Further, an increase in adiposity can result in fat accumulation in depots with different metabolic properties, such as visceral fat or subcutaneous fat, where the former has a higher risk for development of metabolic disease [47]. Reasons proposed to contribute to the risks associated with visceral fat accumulation include the location of an intra-abdominal fat depot in relation to the portal circulation, with a likely direct effect of lipolyzed fatty acids on hepatic metabolic pathways [45]. However, changes in the microenvironment may also contribute to such risks.

Obesity, and in particular visceral obesity, is associated with many metabolic effects including a chronic release of pro-inflammatory cytokines such as tumor necrosis factor (TNF-α). A change in the cytokine balance towards a more pro-inflammatory setting has many potential downstream effects, including stimulation of lipolysis, impaired preadipocyte differentiation, and promotion of monocyte recruitment [36]. In obesity, mature adipose tissue becomes increasingly populated with macrophages, with the release of more inflammatory mediators thus propagating the inflammatory cascade. Two different subpopulations of macrophages have been identified in adipose tissue, the pro-inflammatory classical M1 and anti-inflammatory alternative M2 [43]. The ratio of M1 to M2 has been suggested to influence the metabolic complications of obesity, which may be associated not only with a sustained pro-inflammatory reaction but also with a failure of anti-inflammatory control mechanisms such as IL-10, which has been shown to decrease monocyte activation. Expansion of adipose tissue also results in changes in adipokine signaling, with secondary effects for metabolically active tissues such as the liver, muscle, and the gastrointestinal tract.

Obesity is a cardinal feature of the pro-inflammatory metabolic syndrome representing a constellation of physical and biochemical findings (e.g., central adiposity, hypertension, dyslipidemia, impaired glucose tolerance) with underlying insulin resistance predisposing affected individuals to cardiovascular disease and type 2 diabetes. Additional associated risk factors such as triglyceride-rich lipoproteins, pro-inflammatory cytokines (e.g., IL-1, TNF-α), adhesion molecules (e.g., ICAM-1, selectins), inflammatory stimuli with hepatic effects (e.g., IL-6), and acute phase products resulting from hepatic stimulation (e.g., serum amyloid-A and C-reactive protein [CRP]) all contribute to the pro-inflammatory environment of the syndrome [15,60]. Although the components may differ between individuals, those with metabolic syndrome are at increased cardiovascular risk [61]. The presence of the metabolic syndrome at younger ages is therefore likely to lead to a lifetime burden of inflammation and early risk for chronic disease. Insulin resistance is believed to be a critical pathophysiological event in the disease process, impacting both skeletal muscle metabolic function as well as vascular responses, with reduced skeletal muscle mass, altered insulin signaling, and impaired mitochondrial function.

Even in the absence of obesity, pregnancy is associated with an inflammatory pattern including a significant rise in CRP levels, macrophages, and neutrophils [14]. With placental growth, higher IL-10 levels are produced with a resulting anti-inflammatory effect. Inflammation and inflammatory cytokines have been shown to play a role in placental function regardless of obesity; all known cytokines are synthesized and released by cytotrophoblast, syncytiotrophoblast, and resident placental macrophages [22]. However, obesity may add to a heightened adverse fetal environment. Obese women begin their pregnancies with established chronic inflammation and endothelial activation, and display exaggerated lipid mobilization, a greater degree of insulin resistance, and increased uteroplacental vascular resistance [29]. While healthy pregnant women typically store fat in lower body adipose depots, there is fatty acid excess and ectopic fat accumulation, which is further exaggerated by additional fat accumulation during pregnancy. These findings underscore the importance of the maternal-fetal relationship in the development of an unfavorable milieu for both the fetus and the mother [48]. This synergistic environment is proposed as a key element in establishing negative long-term consequences and the predisposition for a premature aging phenotype. 

## 4. Developmental Adipogenesis and Key Precursors

The presence of excess adiposity may influence fetal development in a variety of ways. The development of adipose tissue is complex with heterogeneity between different adipose tissue fractions, white adipose tissue (WAT) and brown adipose tissue (BAT), as well as within these fractions and between different depot locations. Histologically, five stages of adipose tissue formation have been documented in human fetuses from 5 to 29 weeks gestation, from the early emergence of mesenchymal structures to the formation of definitive fat lobules [62,63]. The first histological evidence of adipose tissue has been shown to occur in Stage 2, when condensation of mesenchyme is associated with vascular proliferation and a rich capillary network around which mesenchymal cells differentiate into preadipocytes. Mesenchymal matrix organization and adipose formation during the second trimester was shown to include evidence of buccal fat pads. Intense fat cell proliferation was noted through 23 weeks gestation whereas from 24 to 29 weeks, the growth of adipose tissue appeared to reflect an increase in fat lobule size. In a subsequent study with more than 400 human specimens, fat lobules were confirmed as the earliest structures identified before the appearance of adipocytes. In the final stages, primitive fat lobules developed into definitive structures resembling those seen in newborns; no differences were observed when comparing males and females. Overall, it was concluded that the second trimester is the critical period for fat development, and by 28 weeks adipose tissue is present in the principal body fat depots. Notably, exposure of the fetus to nutritional and environmental challenges during this formative period could result in epigenetic modifications with lasting effects into adulthood [17,18]. Epigenetic changes during key developmental stages have been reported in animal models and human studies [19,20,21]. Epigenetic modifications are likely to be highly variable and may alter developing adipocytes differently depending on location, temporal relationships, and the microenvironment.

As noted, a prominent feature of fetal adipose histogenesis is a close spatial and temporal relationship between blood vessel and adipocyte development. This close relationship suggests the vascular system may play a role in alterations in adipose tissue properties. Whether vascular development precedes adipocyte development remains unclear although studies suggest vascular development may be dependent on a fat depot [62,63]. These studies have provided insight into early adipose development, but mainly reflect morphology and do not address the earliest determinants of a commitment to an adipogenic lineage. Such information gaps represent important areas for study to further understand the ontogeny of adipose tissue at different anatomical sites. 

## 5. Relationship between BAT and WAT

While white adipocytes have a primary role in triglyceride storage, brown adipocytes serve as a source of inducible energy expenditure in the form of thermogenesis, which occurs through expression of uncoupling protein (UCP)-1, a 32 kDa protein found in the inner mitochondrial membrane [64]. Further, WAT is involved in energy storage and known to increase with advancing age while BAT decreases with maturation in humans, although metabolically active BAT has been shown to persist [37]. BAT has been proposed to emerge earlier in development than WAT [38,39,40], and is found in axillary, cervical, peri-renal, and peri-adrenal sites. BAT is associated with age-related involution, although more recent studies have shown that metabolically active BAT persists [37]. *In vivo* imaging studies have indicated that the distribution of BAT is similar in children when compared to adults, but is much more extensive [37,41,42]. Recent publications suggest that WAT and BAT have a different embryonic origin [38,39]. A lineage-tracing study in mice demonstrated that brown adipocytes arise from a myogenic factor 5-positive lineage with PRDM16 a key regulator of a brown fat/skeletal muscle switch suggesting a common mesodermal origin that differs from WAT [40]. Expression profiling also showed that brown fat cells express a number of muscle-related genes. However, brown fat cells that appeared in WAT as a result of cold exposure did not provide evidence to suggest they were derived from the same progenitors. These findings raise questions regarding whether there are different early lineages, or if there is a more primitive stem/progenitor cell from which both BAT and WAT originate. It is tempting to suggest that a differential fetal metabolic load, due to variability in maternal obesity, could lead to differences in adipose tissue development, resulting in differences in the programming leading to WAT and BAT development. The relative lack of knowledge of the role in obesity underscores the need for a better understanding of early fetal programming mechanisms. Future studies are needed to assess the importance of BAT and the BAT/WAT balance in childhood obesity and the relationship to long-term health outcomes.

## 6. Fetal Environment, Energy Metabolism, and Adiposity

A growing body of research implicates an expansion of adipose tissue and fat infiltration into multiple organs in parallel with an inflammatory burden as contributory to cardiovascular disease, Alzheimer’s disease, diabetes mellitus, and various forms of malignancy. Examples of such fat deposition include non-alcoholic hepatic steatosis and fat infiltration of muscle [49]. This challenging degree of complexity has made it difficult to identify targets for intervention. Current approaches include behavioral interventions based on diet and exercise and a growing use of surgical interventions. While numerous pathways are likely to contribute to these conditions it is interesting to note that a number of studies highlight mitochondrial function as a key factor in regulating energy balance and establishing a critical metabolic set point. As noted, mitochondrial function is a differentiating property between WAT and BAT. Examples of conditions where a reduction in mitochondrial function and/or numbers are suggested as contributory include aging, diabetes mellitus, and HIV infection [54,55]. HIV infection is of particular interest because it is associated with accelerated aging, a concept that is likely to share many similarities with the premature aging phenotype we propose that results from fetal exposure to a lipid-rich environment. In order to fully assess the overall effects of changes in mitochondrial function, the integration of such findings with a comprehensive assessment of metabolic conditions will be required. 

It has been established that maternal triglycerides do not directly cross the placenta but provide a ready source of fatty acids for the fetus. In this context, the presence of diabetes mellitus has been shown to have a profound impact on maternal circulating lipids promoting transplacental transfer by increasing the maternal-fetal concentration gradient particularly for free fatty acids [56]. The continuous and active transfer of nutrients across the placenta is important for fetal metabolism and growth as well as fat deposition. Substrates that cross the placenta in greatest quantity include glucose and amino acids, with free fatty acids and glycerol to a lesser degree [50]. In humans, the placenta has been shown to be relatively permeable to free fatty acids and it has been suggested that during early gestation, embryonic and fetal lipids are derived from maternal free fatty acids, whereas in the third trimester there is a gradual shift to fetal tissue synthesis. However, other metabolic building blocks have the potential to be converted to fat. For example, the rapid accumulation of fat in the third trimester has been linked to maternal glucose levels. The importance of carbohydrates as a source of *de novo* fatty acid formation throughout the lifespan has recently been underscored in studies on the role of fructose, which demonstrates an increase in lipogenesis from fructose compared to glucose [51,52,53]. 

## 7. Conclusions

**Figure 2:**
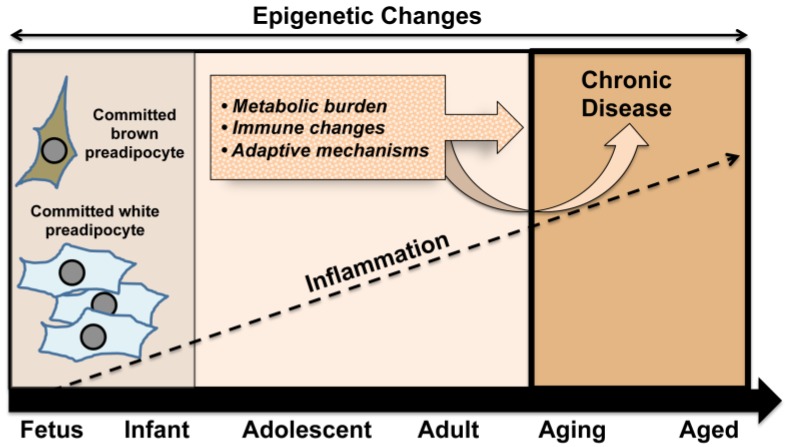
In a mutually reinforcing way, early set points in the developmental process, such as the balance between white and brown adipose tissue, act together with exposures across the lifespan to enhance metabolic burden and immune system changes. In concert with adaptive mechanisms, these conditions underpin an increased likelihood of developing chronic disease in later life.

While optimal nutrition remains an important cornerstone for healthy fetal development, the frequency of maternal obesity at the global level has raised concerns regarding the impact of an obese environment on the developing fetus. An increasing number of reports suggest that obesity and chronic inflammation established prior to the onset of pregnancy can negatively impact the intrauterine environment [65]. This and related events, including a heightened inflammatory setting, may set the stage for conditions with an accumulating burden of chronic disease leading to the proposed premature aging phenotype (Figure 2).

The development of this phenotype sustains the characteristics of adult disease including visceral fat deposition, chronic inflammation, obesity, compensatory modulations, and associated health complications. Early development of chronic disease is likely to represent an increasing burden on the health care system globally, thus a better understanding of underlying mechanisms to inform appropriate preventive measures and interventions is clearly warranted.
